# Dupuytren’s disease is a work-related disorder: results of a population-based cohort study

**DOI:** 10.1136/oemed-2022-108670

**Published:** 2023-01-12

**Authors:** Bente A. van den Berge, Akira Wiberg, Paul M. N. Werker, Dieuwke C. Broekstra, Dominic Furniss

**Affiliations:** 1 Department of Plastic Surgery, University Medical Center Groningen, Groningen, The Netherlands; 2 Nuffield Department of Orthopaedics, Rheumatology and Musculoskeletal Sciences, The Botnar Research Centre, Oxford University, Oxford, UK; 3 Department of Plastic and Reconstructive Surgery, Oxford University Hospitals NHS Trust, Oxford, UK

**Keywords:** Preventive medicine, Occupational Health

## Abstract

**Objectives:**

Dupuytren’s disease (DD) is a fibroproliferative disorder of the hands, characterised by the development of fibrous nodules and cords that may cause disabling contractures of the fingers. The role of manual work exposure in the aetiology of DD is controversial. We investigated whether current occupational exposure to manual work is associated with DD, and if there is a dose–response relationship.

**Methods:**

In this population-based cohort analysis, we used data from the UK Biobank cohort. Our primary outcome was the presence of DD. The exposure of interest was manual work, measured for each participant in two different ways to allow two independent analyses to be undertaken: (1) the current manual work status of the occupation at the time of recruitment, and (2) a cumulative manual work exposure score, calculated based on the occupational history. We performed propensity score matching and applied a logistic regression model.

**Results:**

We included 196 265 participants for the current manual work analysis, and 96 563 participants for the dose–response analysis. Participants whose current occupation usually/always involved manual work were more often affected with DD than participants whose occupation sometimes/never involved manual work (OR 1.29, 95% CI 1.12 to 1.49, p<0.001). There was a positive dose–response relationship between cumulative manual work exposure score and DD. Each increment in cumulative work exposure score increased the odds by 17% (OR 1.17, 95% CI 1.08 to 1.27, p<0.001).

**Conclusions:**

Manual work exposure is a risk factor for DD, with a clear dose–response relationship. Physicians treating patients should recognise DD as a work-related disorder and inform patients accordingly.

**WHAT IS ALREADY KNOWN ON THIS TOPIC**

- Dupuytren’s disease is a fibroproliferative disorder of the hands, characterised by the development of fibrous nodules and cords that may cause disabling contractures of the fingers. The role of manual work exposure in the disease aetiology is still under debate, and clarity around this relationship is important for patients, physicians and industrial compensation schemes worldwide.

**WHAT THIS STUDY ADDS**

- Our study provides strong evidence that manual work exposure is a risk factor for Dupuytren’s disease, with a clear dose–response relationship.

**HOW THIS STUDY MIGHT AFFECT RESEARCH, PRACTICE OR POLICY**

- Physicians and surgeons treating patients should recognise Dupuytren’s disease as a work-related disorder and should inform patients accordingly.

## Introduction

Dupuytren’s disease is a common connective tissue disorder of the hand. Patients develop fibrous nodules in the palm of the hand, which can evolve into cords which may contract, causing one or more fingers to be pulled into a flexed position. The disease is present in up to 31.6% of the general population, and men are more frequently affected than women.^1^ Although the disease was named after French surgeon Baron Dupuytren who famously operated on his wine merchant, Sir Henry Cline of London was one of the first to give an appropriate description of the affection in 1777. He addressed the palmar fibrosis as a disease of ‘laborious people’.^2^ Nowadays, there is consensus about certain risk factors for Dupuytren’s disease, such as smoking, alcohol intake, diabetes mellitus (DM) and genetic predisposition,^3 4^ but the role of manual work exposure in the disease aetiology remains unclear.

Several studies on the association between manual work exposure and Dupuytren’s disease show conflicting results.^5–9^ Although most publications on this subject report a positive association between manual work and Dupuytren’s disease,^3^ the majority of these studies were limited by a highly selected or small study population, and the lack of adjustment for important confounders.^10^ Obtaining a definitive answer to the question of whether Dupuytren’s disease is an occupational disease would give us more insight into its aetiology, and would be of great value in informing preventative strategies.

In this population-based cohort study, we aimed to investigate whether occupational exposure to manual work is associated with Dupuytren’s disease, and whether a dose–response relationship is present.

## Methods

### Study design

We used data from the UK Biobank, a large nationwide population-based prospective cohort study established to allow investigation of determinants of the diseases of middle and old age.^11^ Between 2006 and 2010, approximately 500 000 participants aged between 40 and 69 years old were recruited from the general population at 22 assessment centres throughout the UK. A wide variety of exposures and health outcomes were collected with self-completed questionnaires, a computer-assisted interview, functional and physical measures, and collection of blood, urine, and saliva. Participants also consented to linkage of these data with their hospital medical records. All participants provided written informed consent for the use of their data for this research. More details about the full UK Biobank cohort can be found at www.ukbiobank.ac.uk/.

### Participants

Because Dupuytren’s disease is most often reported in people of North-Western European descent,^1 12^ we selected participants whose self-reported ethnicity was ‘white British’, confirmed by genetic principal component analysis.^13^ The exclusion criteria and the quality control have been described in detail in a previous study using the same curated version of the raw UK Biobank data.^13^ After participant withdrawal at the time of data extraction for the current study, the overall study population comprised 401 573 participants.

### Outcome

The primary outcome measure was the presence of Dupuytren’s disease. Participants with Dupuytren’s disease were identified by selecting individuals who had one of the following diagnosis and surgery codes (online supplemental table 1):

10.1136/oemed-2022-108670.supp1 Supplementary data

1. International Classification of Diseases (ICD-10) code for Dupuytren’s disease.
2. Office of Population Censuses and Services-4 code for Dupuytren’s surgery.
3. Self-reported Dupuytren’s disease code at recruitment.
4. Self-reported Dupuytren’s surgery code at recruitment.

### Variables

#### Manual work exposure

The exposure of interest was manual work. We measured the manual work exposure in two different ways to allow two independent analyses to be undertaken: (1) the current manual work status of the participant’s current occupation at the time of recruitment, and (2) a cumulative manual work exposure score for each participant, calculated based on their occupational history.

1. Current manual work status: at recruitment, UK Biobank participants who were still in employment reported their manual work status by answering whether their current job involves manual or physical work (‘rarely’, ‘sometimes’, ‘usually’, or ‘always’).
2. Cumulative manual work exposure: trained UK Biobank staff conducted a verbal interview to register the participants’ current or most recent occupation according to the UK Standard Occupational Classification (SOC) 2000 system.^14^ In 2015, UK Biobank participants were asked to complete their complete occupational history according to the UK SOC 2000. Each job included in the job history lasted at least six months, for at least 15 hours a week.

To characterise the manual work requirements of each occupation, we used the Occupational Information Network (O*NET).^15^ The O*NET dataset contains the physical requirements of almost 1000 occupations, defined by the SOC classification. Job demand data in O*NET are derived from questionnaires of professionals, workers and job analysts familiar with each job. Because O*NET uses the US 2010 version of the SOC classification,^16^ we used a job exposure matrix (JEM) for use in the UK Biobank^17^ to translate the UK SOC codes to US SOC codes. We identified three questions from the O*NET database about manual work exposure in relation to their job: ‘How important is handling and moving objects to the performance of your job?’ (Likert scale 1–5), ‘What level of handling and moving objects is needed to perform your job?’ (Likert scale 0–7), and ‘How much time in your job do you spent using your hand to handle, control and feel objects, tools or controls?’ (Likert scale 1–5). We gave each question equal weighting, standardised the score of each question to a scale ranging from 0 to 100, and calculated the mean manual work exposure score for each occupation. Several common occupations and their associated O*NET scores are shown in table 1.

**Table 1:** Examples of occupations and corresponding O*NET scores for manual exposure

O*NET- score	Occupation
0–10	Management analysts, mediators, epidemiologists
10–20	Financial advisors, marketing managers, social workers, advertising sales agents
20–30	Accountants, lawyers, dietitians, security guards, computer programmers
30–40	Landscape architects, concierges, telephone operators, commercial and industrial designers
40–50	Biologists, librarians, nurse practitioners, radio operators, occupational therapists
50–60	Lifeguards, office machine operators, pharmacists, obstetricians and gynaecologists, bakers
60–70	Surgeons, photographers, subway operators, waiters/waitresses, cooks, shoe repairers
70–80	Barbers, electrical repairers, aircraft mechanics and service technicians, crane operators
80–90	Fishers, industrial truck and tractor operators, cabinetmakers and bench carpenters, roofers
90–100	Stonemasons, pipelayers, carpet installers

We then determined the participants’ manual work exposure for each job by multiplying the O*NET score for that job by the number of years in that job, thus allowing us to calculate an individual cumulative manual work exposure score by the sum of all the exposure scores of each participant. A flow chart of the steps involved in this analysis is shown in online supplemental figure 2.

10.1136/oemed-2022-108670.supp2 Supplementary data

#### Other variables

We collected the following baseline characteristics that are potential confounders previously associated (weakly or strongly) with DD for the association between DD and manual work exposure^3 18^: age at inclusion, sex, DM, hypertension, respiratory disease, smoking status, alcohol intake, body mass index (BMI), cholesterol levels (low-density lipoprotein (LDL) and high-density lipoprotein (HDL)), triglyceride levels and Townsend Deprivation Index (TDI).^19^ Age at recruitment was defined as the age in 2008—the halfway point of the 4-year recruitment period. The presence of DM, hypertension and respiratory disease was identified through patient self-report, and with ICD-10 diagnostic codes (online supplemental table 1). Self-reported smoking status was recorded as ‘previous or current use’, ‘never’ and ‘prefer not to answer’. Self-reported alcohol intake frequency was recorded as: ‘daily or almost daily’, ‘three or four times a week’, ‘once or twice a week’, ‘one to three times a month’, ‘special occasions only’, ‘never’ and ‘prefer not to answer’. BMI was calculated with the weight and height measured by trained staff at recruitment. Cholesterol and triglyceride levels were measured at baseline. TDI was determined with national census data linked to the participants postcode: the greater the TDI, the greater the degree of deprivation. A high TDS reflects low socioeconomic status and is associated with low self-reported health^20^ and an unhealthy lifestyle, including physical activity and diet.^21^ Hence, the TDI complements to the other health-related variables included in the analysis. Further information on the UK Biobank data is described in more detail elsewhere.^11^

### Data processing and statistical analyses

Baseline characteristics were reported by means and SDs for normally distributed continuous variables and by medians and IQRs for non-normally distributed continuous variables. For dichotomous variables, frequencies and proportions were reported.

#### Analysis 1: current manual work

To study the association between current manual work and Dupuytren’s disease, we first performed propensity score matching (PSM) to balance the groups.^22^ We excluded all participants with a missing current manual work status and with a missing value in one of the risk factors included in the PSM, since no propensity score could be calculated for them.

Propensity scores were calculated through logistic regression modelling based on the following covariates: age, sex, DM, hypertension, respiratory disease, smoking status, alcohol intake, BMI, LDL, HDL, triglyceride levels and TDI. We applied the nearest neighbour method without replacement and set a calliper width at 0.2 of the logit of SD of the propensity score. We identified 1:4 matching of exposed to unexposed participants as the optimal matching ratio, based on the balance diagnostics after matching.^23^

We matched participants whose current job ‘usually’ or ‘always’ involves manual work (exposed) with participants whose current job ‘rarely’ or ‘sometimes’ involves manual work (unexposed), based on the participants’ propensity score. We applied a logistic regression model with the binarised manual work status as the exposure, and Dupuytren’s disease as the outcome. We evaluated the need to add a random intercept for each matched set of exposed and unexposed participants, to take clustering into account that may have been introduced in the dataset because of the matching procedure. This clustering was very low, so we implemented a model without random effects.

#### Analysis 2: dose–response relationship

We investigated whether a greater cumulative amount of manual work is associated with a higher risk of having Dupuytren’s disease. For this dose–response analysis, we excluded participants with missing manual work status, missing data on any of the risk factors included in the PSM, and participants with missing occupational data. Additionally, we excluded participants with a total occupational history of <15 years or >55 years, because all participants were aged between 40 and 69 years at the time of recruitment, making it unlikely that they were employed for less than 15 years or longer than 55 years. We also excluded participants with overlapping years of jobs in their job history, because there was a risk of input error. There was a linear relationship between the cumulative manual work exposure score and the logit of proportion of Dupuytren’s disease patients (online supplemental figure 2). To ease the interpretation of our results, we categorised the cumulative work exposure scores into five groups separated by increments of 750, as follows: <750, 750–1499, 1500–2249, 2250–3000, >3000 standardised O*NET-score×years.

We applied the same PSM model we used in the current manual work analysis. Again, we matched participants whose current job ‘usually’ or ‘always’ involves manual work (exposed) with participants whose current job ‘rarely’ or ‘sometimes’ involves manual work (unexposed). After PSM, we applied a logistic regression model with the cumulative manual work exposure score as the exposure and Dupuytren’s disease as the outcome. Again, we evaluated the necessity for a random intercept, but this was not required. A two-sided p value<0.05 was considered statistically significant. All analyses were carried out using R V.4.1.3,^24^ the *MatchIt* 25 and *lme4* 26 packages.

### Sensitivity analyses

To check the robustness of our results with PSM, we used a multivariable logistic regression model without matching as a sensitivity analysis, with Dupuytren’s disease as the outcome variable. For both sensitivity analyses, we adjusted for the same explanatory variables we included in the propensity score model. For the current manual work sensitivity analysis, we additionally included the binarised manual work status as explanatory variable. For the dose–response sensitivity analysis, we included the cumulative manual work exposure score as explanatory variable. A two-sided p value<0.05 was considered statistically significant.

## Results

### Participants

**Figure 1:**
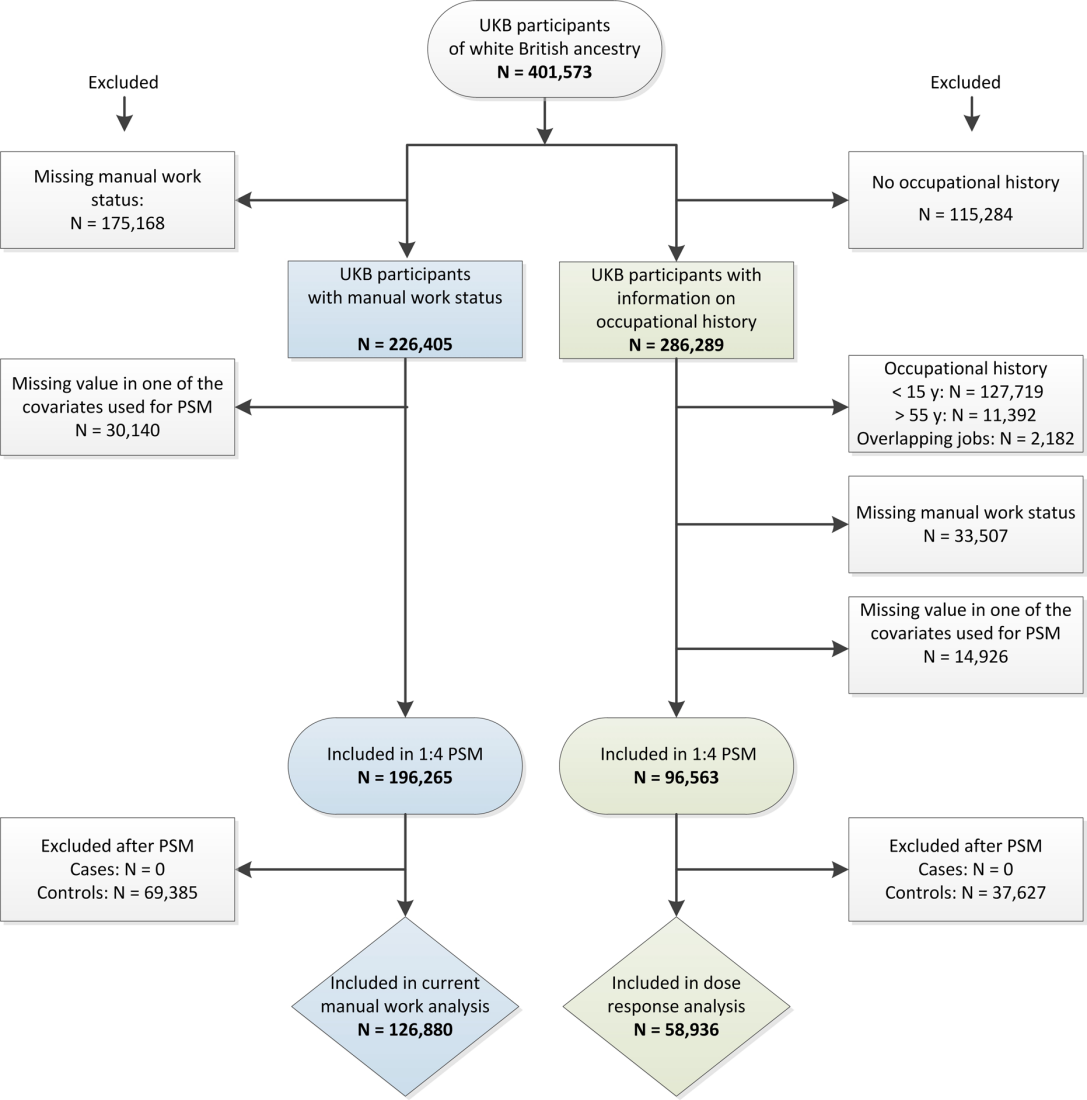
Flowchart of the participant selection process.

After applying our exclusion criteria, we identified 196 265 eligible participants for the current manual work analysis, and 96 563 for the dose–response analysis (figure 1). After PSM, we included 126 880 participants (26 667 exposed and 100 213 unexposed) in the current manual work analysis, and 58 936 participants (12 312 exposed and 46 624 unexposed) in the dose–response analysis. All exposed participants could be matched to at least one unexposed participant.

In the original study population of the current manual work analysis, the mean age was 53 years in both the exposed and unexposed groups. Exposed participants were more often male (63.5%) and smokers (49.1%), compared with unexposed participants (46.6% and 41.7%). In the original study population of the dose–response analysis, the mean age was 53 years in both the exposed and unexposed groups. Exposed participants were more often male (66.5%) and smokers (46.6%), compared with unexposed participants (46.7% and 39.3%). In the matched population of the current manual work analysis, 259 (0.97%) exposed and 755 (0.75%) unexposed participants had Dupuytren’s disease (table 2). In the matched population of the dose–response analysis, 114 (0.93%) exposed and 382 (0.82%) unexposed participants had Dupuytren’s disease. After PSM, the distribution of the covariates was balanced between the exposed and unexposed groups in both study populations (table 2). The balance diagnostics of all covariates are shown in more detail in online supplemental figure 2.

**Table 2:** Descriptive statistics of the study population of the current manual work analysis and the dose–response analysis, before and after propensity score matching

Manual work status	Current manual work analysis						Dose–response analysis					
	Unmatched			Matched			Unmatched			Matched		
	Exposed (usually/always)	Unexposed (rarely/never)	SMD	Exposed (usually/always)	Unexposed (rarely/never)	SMD	Exposed (usually/always)	Unexposed (rarely/never)	SMD	Exposed (usually/always)	Unexposed (rarely/never)	SMD
N	26 667	169 598		26 667	100 213		12 312	84 251		12 312	46 624	
Age, mean (SD)	52.73 (7.15)	52.98 (7.07)	−0.035	52.73 (7.15)	52.81 (7.18)	−0.001	53.27 (7.07)	53.26 (6.91)	0.001	53.27 (7.07)	53.30 (7.01)	0.002
Sex, m (%)	16 943 (63.5)	79 115 (46.6)	0.353	16 943 (63.5)	61 593 (61.5)	0.001	8187 (66.5)	39 350 (46.7)	0.419	8187 (66.5)	30 259 (64.9)	0.004
DM (%)	1225 (4.6)	6960 (4.1)	0.023	1225 (4.6)	4592 (4.6)	0.004	549 (4.5)	3142 (3.7)	0.035	549 (4.5)	2031 (4.4)	0.002
Smoker (%)	13 087 (49.1)	70 713 (41.7)	0.148	13 087 (49.1)	47 537 (47.4)	0.005	5740 (46.6)	33 134 (39.3)	0.146	5740 (46.6)	20 992 (45.0)	0.006
Alcohol (%)												
(*Never*)	1564 (5.9)	7800 (4.6)	0.054	1564 (5.9)	5480 (5.5)	<0.001	714 (5.8)	3774 (4.5)	0.057	714 (5.8)	2534 (5.4)	0.001
(*Special occasions only*)	2971 (11.1)	14 598 (8.6)	0.081	2971 (11.1)	10 287 (10.3)	0.008	1231 (10.0)	7005 (8.3)	0.056	1231 (10.0)	4357 (9.3)	0.008
*(1–3 times a month*)	3361 (12.6)	19 622 (11.6)	0.031	3361 (12.6)	12 447 (12.4)	<0.001	1468 (11.9)	9311 (11.1)	0.027	1468 (11.9)	5410 (11.6)	0.003
*(1–2 times a week*)	8320 (31.2)	47 166 (27.8)	0.073	8320 (31.2)	30 857 (30.8)	<0.001	3782 (30.7)	23 187 (27.5)	0.069	3782 (30.7)	14 090 (30.2)	0.002
*(3–4 times a week*)	5811 (21.8)	44 771 (26.4)	−0.112	5811 (21.8)	22 779 (22.7)	−0.001	2821 (22.9)	22 882 (27.2)	−0.101	2821 (22.9)	11 119 (23.8)	−0.003
((*Almost) daily*)	4640 (17.4)	35 641 (21.0)	−0.096	4640 (17.4)	18 363 (18.3)	−0.006	2296 (18.6)	18 092 (21.5)	−0.074	2296 (18.6)	9114 (19.5)	−0.007
Triglyc. (mmol/l), mean (SD)	1.78 (1.10)	1.70 (1.02)	0.074	1.78 (1.10)	1.77 (1.06)	0.002	1.77 (1.09)	1.68 (1.01)	0.089	1.77 (1.09)	1.77 (1.06)	−0.003
LDL (mmol/l), mean (SD)	3.59 (0.83)	3.57 (0.83)	0.024	3.59 (0.83)	3.59 (0.84)	<0.001	3.60 (0.83)	3.58 (0.83)	0.034	3.60 (0.83)	3.60 (0.84)	0.006
HDL (mmol/l), mean (SD)	1.41 (0.36)	1.45 (0.37)	−0.112	1.41 (0.36)	1.41 (0.37)	0.006	1.41 (0.36)	1.46 (0.38)	−0.145	1.41 (0.36)	1.41 (1.37)	0.005
BMI, mean (SD)	27.63 (4.59)	27.21 (4.69)	0.090	27.63 (4.59)	27.55 (4.73)	0.002	27.59 (4.49)	27.05 (4.64)	0.121	27.59 (4.49)	27.49 (4.70)	0.006
Hypertension (%)	7129 (26.7)	40 721 (24.0)	0.062	7129 (26.7)	26 361 (26.3)	0.001	3257 (26.5)	20 002 (23.7)	0.062	3257 (26.5)	12 162 (26.1)	<0.001
TDI, mean (SD)	−0.90 (3.07)	−1.70 (2.80)	0.261	−0.90 (3.07)	−1.14 (3.01)	−0.011	−1.15 (2.97)	−1.83 (2.72)	0.229	−1.15 (2.97)	−1.35 (2.91)	0.013
Respiratory disease (%)	3795 (14.2)	24 095 (14.2)	−0.001	3795 (14.2)	14 189 (14.2)	−0.001	1684 (13.7)	11 732 (13.9)	0.007	1684 (13.7)	6377 (13.7)	−0.001

Because participants with missing values were excluded before PSM, there was no missingness of baseline covariates.

BMI, body mass index; DM, diabetes mellitus; HDL, high-density lipoprotein; LDL, low-density lipoprotein; SMD, standardised mean difference between exposed and unexposed participants; TDI, Townsend Deprivation Index; Triglyc., triglycerides.

**Figure 2:**
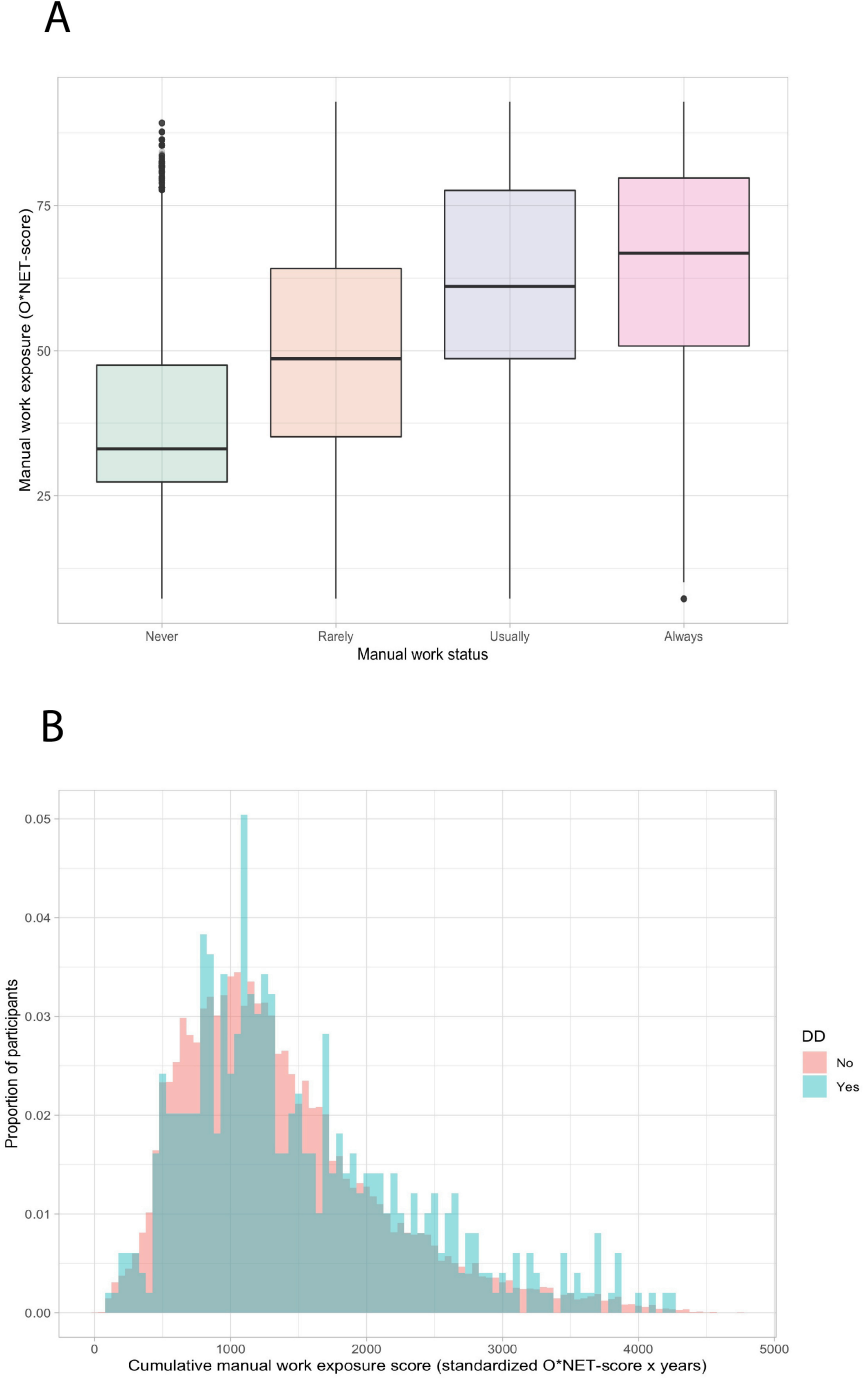
(A) The relationship between the self-reported manual work status and O*NET score (0–100) of each job. The x-axis represents the self-reported manual work status, and the y-axis represents the manual work exposure score (0–100). (B) Cumulative manual work exposure scores of patients with and without Dupuytren’s disease (DD). The y-axis represents the proportion of participants, and the x-axis represents the cumulative work exposure score, grouped into bins of 50 points (standardised O*NET-score×years).

The median length of reported employment was 30 years (IQR 21–38). The O*NET scores of all jobs ranged from 1 (eg, mediators) to 92 (eg, stonemasons) on the standardised 0–100 scale. There is a relationship between the current manual work exposure for the participants’ current job and the O*NET score for that job, indicating that a higher O*NET score is associated with a higher manual work exposure (figure 2A). The median cumulative manual work exposure was 1225 O*NET×years (IQR 838–1744). The distribution of the cumulative manual work exposure is shown in figure 2B. A high cumulative manual work exposure score is more common among Dupuytren’s patients than among participants without Dupuytren’s disease.

### Current manual work exposure

Exposure to manual work in current employment (usually/always vs. rarely/never) was associated with the presence of Dupuytren’s disease (OR 1.29, 95% CI 1.12 to 1.49, p=3.9 ×10^–4^). The OR is a good estimate of the population relative risk (RR) and can be interpreted as a RR.^27^ The results of the sensitivity analysis were very similar (OR 1.26, 95% CI 1.10 to 1.45, p=0.001) (table 3).

**Table 3:** Results of the adjusted multivariable regression analyses (sensitivity analyses) including the current manual work status and the cumulative manual work exposure

Predictors	Current manual work analysis			Dose–response analysis		
	DD			DD		
	OR	95% CI	P value	OR	95% CI	P value
Manual work status	1.26	1.10 to 1.45	**0.001**	*NA*	*NA*	*NA*
Cumulative manual work exposure	*NA*	*NA*	*NA*	1.08	1.01 to 1.16	**0.029**
Age	1.07	1.06 to 1.08	**<0.001**	1.06	1.05 to 1.07	**<0.001**
Sex (m)	3.51	3.05 to 4.04	**<0.001**	3.00	2.48 to 3.65	**<0.001**
DM (y)	2.09	1.71 to 2.53	**<0.001**	2.39	1.81 to 3.11	**<0.001**
Smoker (y)	1.11	0.99 to 1.24	0.064	1.05	0.90 to 1.22	0.506
*Alcohol intake*						
(Never)	1.16	0.83 to 1.60	0.374	0.90	0.54 to 1.44	0.658
(Special occasions only)	0.98	0.73 to 1.32	0.915	1.00	0.66 to 1.50	0.995
(1–3 times a month)	1.10	0.88 to 1.38	0.416	1.04	0.77 to 1.44	0.801
(1–2 times a week) (ref)						
(3–4 times a week)	1.25	1.01 to 1.57	**0.046**	1.25	0.93 to 1.72	0.151
(Almost daily)	1.48	1.19 to 1.86	**<0.001**	1.54	1.14 to 2.11	**0.006**
*Metabolic factors*						
Triglycerides	1.06	1.00 to 1.12	**0.038**	1.08	1.00 to 1.17	0.050
LDL	1.02	0.96 to 1.09	0.507	0.98	0.89 to 1.08	0.682
HDL	1.80	1.51 to 2.14	**<0.001**	1.48	1.15 to 1.90	**0.002**
BMI	0.94	0.93 to 0.96	**<0.001**	0.94	0.92 to 0.96	**<0.001**
Hypertension	1.23	1.09 to 1.38	**0.001**	1.23	1.04 to 1.46	**0.017**
TDI	1.01	0.99 to 1.03	0.489	1.00	0.98 to 1.03	0.797
Respiratory disease	1.07	0.92 to 1.24	0.361	1.09	0.88 to 1.33	0.428

DD, Dupuytren’s disease; LDL, low-density lipoprotein; HDL, high-density lipoprotein; BMI, body mass index; DM, diabetes mellitus; TDI, Townsend Deprivation Index.

### Dose–response relationship

Our results demonstrate a positive dose–response relationship between O*NET scores and Dupuytren’s disease, with each 750-point (standardised O*NET-score×years) increment in cumulative work exposure score increasing the odds of Dupuytren’s disease by 17% (OR 1.17, 95% CI 1.08 to 1.27, p=1.9×10^–4^). Since the prevalence of Dupuytren’s in our population is low (~1.0%), again the OR is a good estimate of the population RR and can be interpreted as a RR.^27^ To illustrate our results by way of examples, an individual who has been a stonemason for 30 years (cumulative exposure score of 30×92 = 2760), is 17% more likely to have Dupuytren’s disease than someone who has been a cook for 30 years (30×67 = 2010), 37% more likely than a general practitioner of 30 years (30×42 = 1260), and 60% more likely than a social worker of 30 years (30×16 = 480). Again, the results of the sensitivity analysis were similar (OR 1.08 95% CI 1.01 to 1.16, p=0.029) (table 3).

## Discussion

We found that manual work was associated with the Dupuytren’s disease, and that an increasing level of manual work exposure is associated with an increasing risk of having Dupuytren’s disease.

Our results show that individuals whose occupation usually or always involves manual work are more often affected with Dupuytren’s disease than individuals whose occupation sometimes or never involves manual work. Since the very first descriptions of Dupuytren’s disease in the 18th century, doctors have speculated that it is a disease that occurs in people exposed to strenuous manual work. In the past decades, several studies have been conducted to test this hypothesis. Most reported a positive association between manual work and DD,^5 7 9 28–31^ but several did not.^6 8 32 33^ Many of these studies were inadequately powered and were performed in highly selected study populations (eg, only in men). Our findings are supported by a smaller population-based analysis by Fadel *et al*,^5^ although in that study the authors included a limited number of potential confounders and only included patients who had had surgery for Dupuytren’s disease.

We found that the positive association between manual work and Dupuytren’s disease followed a dose–response relationship, which means that an increase in manual work exposure is accompanied by an increased risk of Dupuytren’s disease. Our findings are consistent with previous results derived from different methodological approaches.^5 7 9 28 30 31^ These studies have in common that the dose–response relationship was investigated by including manual work exposure as a categorised variable of self-reported manual work exposure. In our analysis, we derived a novel cumulative exposure to manual work score by multiplying an objective manual exposure score for each job by the number of years in that job, a similar concept to “pack-years” of smoking exposure. Hence, we can conclude not only that there is a dose–response relationship between manual work and Dupuytren’s disease, but also that this relationship is linear.

The role of manual activities in the pathophysiology of Dupuytren’s disease is not yet fully understood. There is consensus that Dupuytren’s disease is a result of a localised inflammatory process, in which highly contractile myofibroblasts are induced to proliferate and differentiate by cytokines produced by local immune cells.^34^ Both genetic predisposition and certain non-genetic risk factors, such as age, sex, smoking, alcohol consumption, diabetes and hypercholesterolaemia, play a role in the development and/or progression of the disease.^35^ It has been proposed that mechanical stress in the palmar fascia could lead to local changes in the microvascular endothelium and subsequent microvascular ischaemia,^36^ leading to the production of oxygen-free radicals, which in turn promote the differentiation from fibroblasts to myofibroblasts.^37^ Nonetheless, the exact causal relationship between mechanical stress and Dupuytren’s disease remains incompletely understood and requires further research.

There was a considerable number of missing values in the variables that we included in the PSM. However, the PSM yielded a very good balance in both analyses (maximum SMD of<0.01). Therefore, we reasoned that multiple imputation of the missing values could not further improve the matching results, and therefore has no implications for the robustness of our findings.

One limitation of our study is that we used self-reported job history, which may be prone to recall bias. However, since the cumulative manual work exposure was derived from participants’ occupational history obtained by trained UK Biobank staff and the O*NET JEM, it is unlikely that recall bias would have meaningfully affected the findings of our study. The downside of the use of JEMs is that all workers with the same job title are allocated with the same exposure estimates while there can be substantial interindividual variability within jobs.^38^

Second, we would ideally have included the effect of manual activities exposure during leisure in addition to the occupational exposure in our analyses.^39^ However, because these data were not available to us in the UK Biobank cohort, we were not able to determine the full exposure to manual activities for each participant. It is known that individuals with more physically demanding jobs, on average, have a lower social economic status, while higher socioeconomic groups tend to undertake more physically demanding leisure activities than lower socioeconomic groups.^40^ We attempted to mitigate any potential bias arising from socioeconomic disparities by including the TDI and other variables related to socioeconomic status (BMI, smoking and alcohol consumption^41 42^) in our matching algorithm. Third, it has been previously reported that, besides manual work, hand transmitted vibration (HTV) is a risk factor for Dupuytren’s disease. Because information on HTV was unavailable in both UKB-data and O*NET-data, we were unable to include HTV in our analyses. In theory, this could have led to an overestimation of the effect of manual work. Lastly, we only included participants of White-British ethnicity. Therefore, our findings and conclusions should be limited to this specific population.

The main strength of our study is the substantial size of the UK Biobank cohort. Second, we undertook two separate analyses to address the research question and confirmed each with a sensitivity analysis using different statistical methodology. All previous studies with a similar aim to ours used a univariable or multivariable regression model. However, it has been emphasised in multiple methodological studies that regression adjustment is not able to remove all the bias if the covariate baseline distributions differ widely, and that PSM is preferred if the means of the propensity scores of exposed and unexposed are more than ½ SD apart,^43^ which was the case in both our analyses. We have also considered inversed probability weighting (IPW), as PSM could lead to exclusion of unmatched participants. However, in our analyses, all exposed cases could be matched with at least one unexposed case because of our large dataset. Besides, PSM has the small benefit compared with IPW in that it causes a slightly larger reduction of bias.^44^ Third, our effect estimates are considerably more precise than estimates reported in previous studies, because we were able to include more important confounders and independent risk factors compared with previous studies. Moreover, we included multiple continuous variables (BMI, serum lipid levels and TDI) in our model, which allowed us to isolate the effect of manual work more accurately than if using only categorical variables. Finally, by using a JEM, we could translate the manual work exposures in a systematic and unbiased way.

Our study shows that manual work exposure is a risk factor for Dupuytren’s disease, with a clear linear dose–response relationship. Hence, physicians and surgeons treating patients should recognise Dupuytren’s disease as a work-related disorder and should inform patients accordingly. Employers could consider interventions to limit manual work exposure, especially in those workers otherwise predisposed to the development of DD. Manual work exposure can be included in future prediction models to estimate the risk for developing Dupuytren’s disease. Further research is needed to elucidate the underlying pathophysiological mechanism.

## Data Availability

Data may be obtained from a third party and are not publicly available. Full UK Biobank data are available by direct application to UK Biobank.
