# Supplementary material for: Dupuytren’s disease is a work-related disorder: results of a population-based cohort study
Source: Occup Environ Med. 2023 Jan 12;80(3):137–45. doi: 10.1136/oemed-2022-108670 (PMC9985760; doi:10.1136/oemed-2022-108670)
Supplement: Supplementary data [file oemed-2022-108670supp001.pdf]

Supplementary Table 1. Codes used to identify exposures and outcome

Exposure	UKBiobank data-fields	OPCS code[1]	ICD-10 code[2]	Non-cancer illness code[3]	Operation code[4]
Dupuytren's disease	-	T521, T522, T561, T562, T525, T526	M72.0	1544	1535
Current manual work status	816				
Age	34	-	-	-	-
Sex	22001	-	-	-	-
BMI	21001	-	-	-	-
Diabetes Mellitus	2443	-	E10, E11, E13, E14, G59.0, G63.2, H28.0, H36.0, M14.2, N08.3, T38.3, Y42.3, O24.0, O24.1, O24.2, O24.3	1220, 1222, 1223, 1607, 1276, 1468	-
Triglycerides	30870	-	-	-	-
HDL Cholesterol	30760	-	-	-	-
LDL Cholesterol	30780	-	-	-	-
Smoking	20116	-	-	-	-
Hypertension	-	-	I10, I11, I12, I13, I14, I15	1065, 1072	-
Townsend Deprivation Index	189	-	-	-	-
Respiratory disease	-	J40, J41, J42, J43, J44, J45, J46, J47, J84	-	1111, 1112, 1113, 1114, 1115, 1121, 1122	-

[1] OPCS code UK Biobank data-field: 41272
[2] ICD-10 code UK Biobank data-field: 41270
[3] Non-cancer illness code: Self-report, UK Biobank data-field: 41272
[4] Operation code: Self-report, UK Biobank data-field: 41272
