# Supplementary material for: Dupuytren’s disease is a work-related disorder: results of a population-based cohort study
Source: Occup Environ Med. 2023 Jan 12;80(3):137–45. doi: 10.1136/oemed-2022-108670 (PMC9985760; doi:10.1136/oemed-2022-108670)

Supplementary figure 1: Flowchart of the steps involved in the dose-response analysis

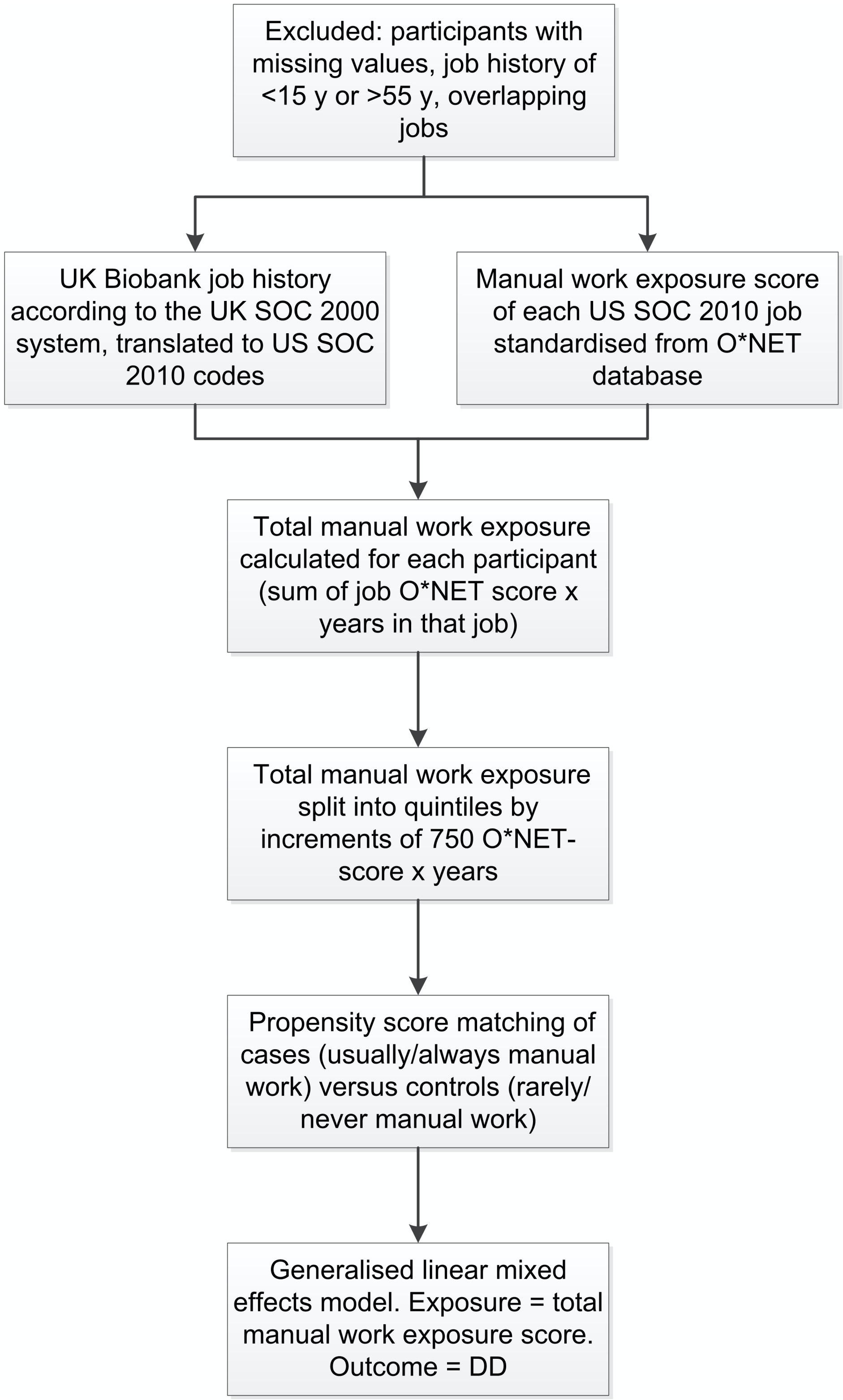

Supplementary Figure 2: the relationship between Dupuytren's disease and manual work exposure

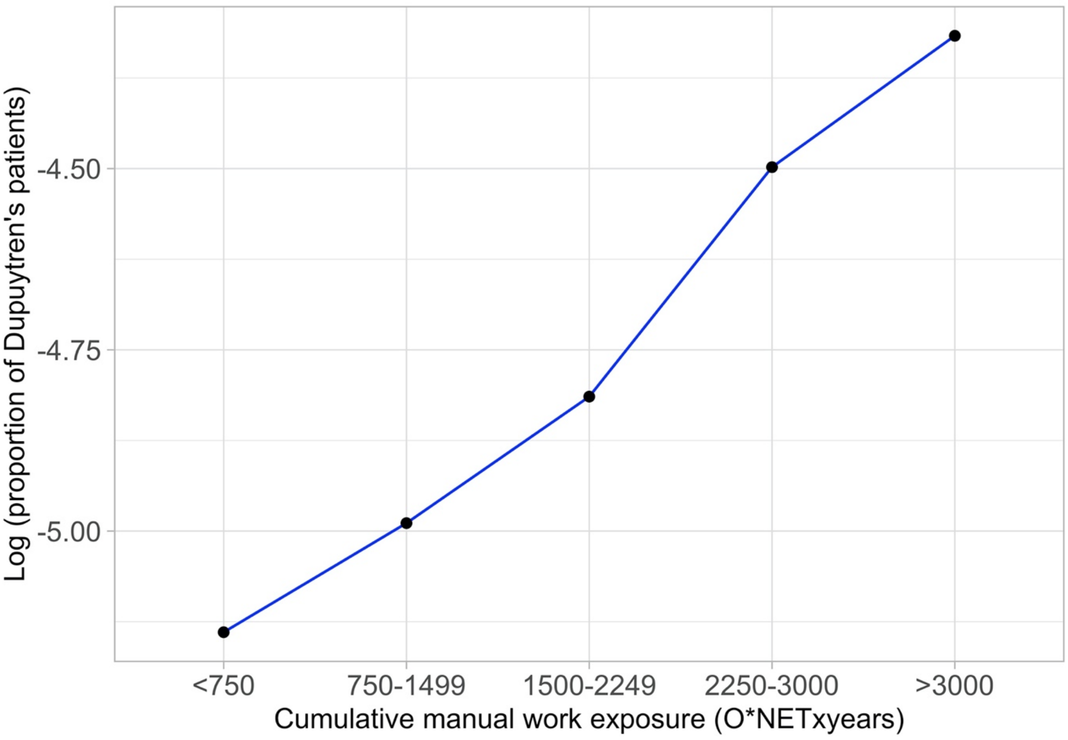

Supplementary figure 3a: Covariate balance before and after matching (current manual work response analysis)

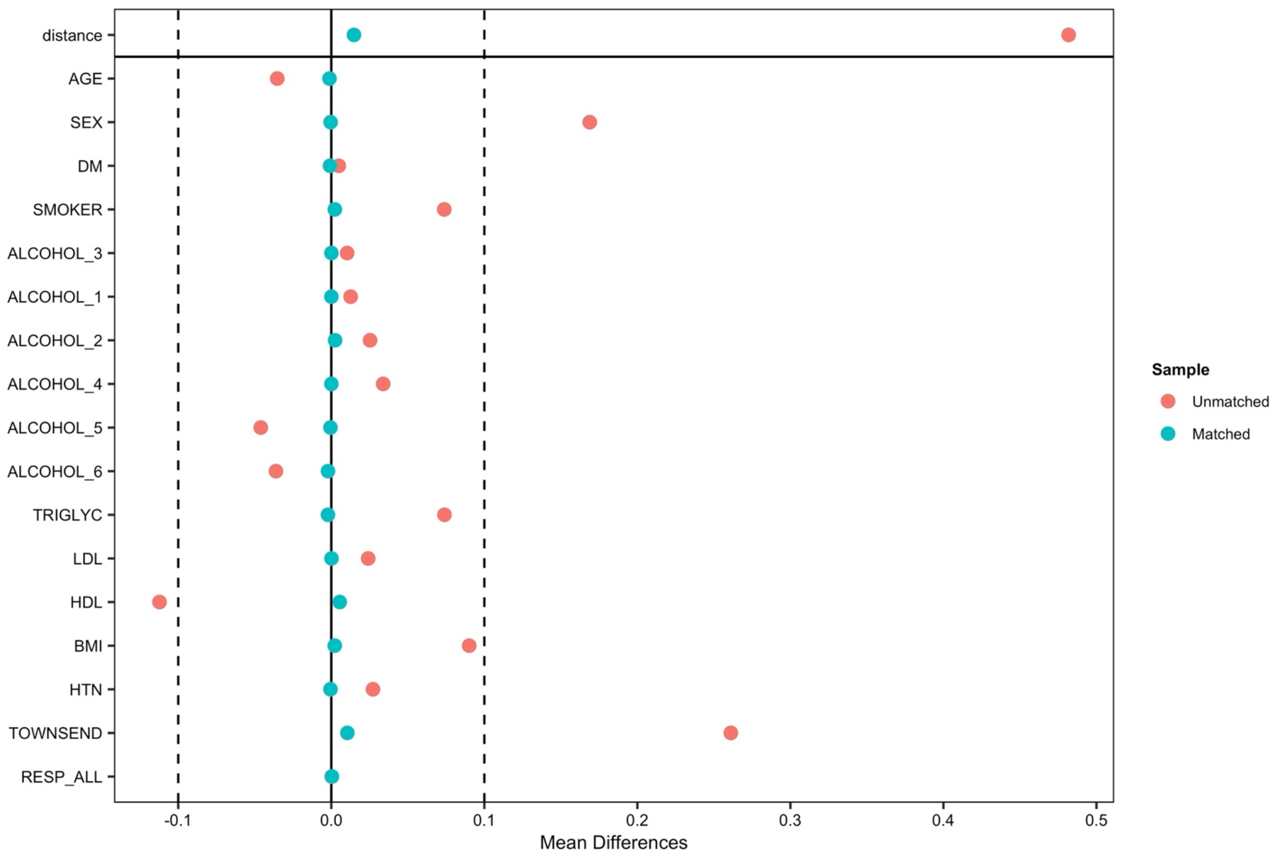

Supplementary figure 3b: Covariate balance before and after matching (dose-response analysis)

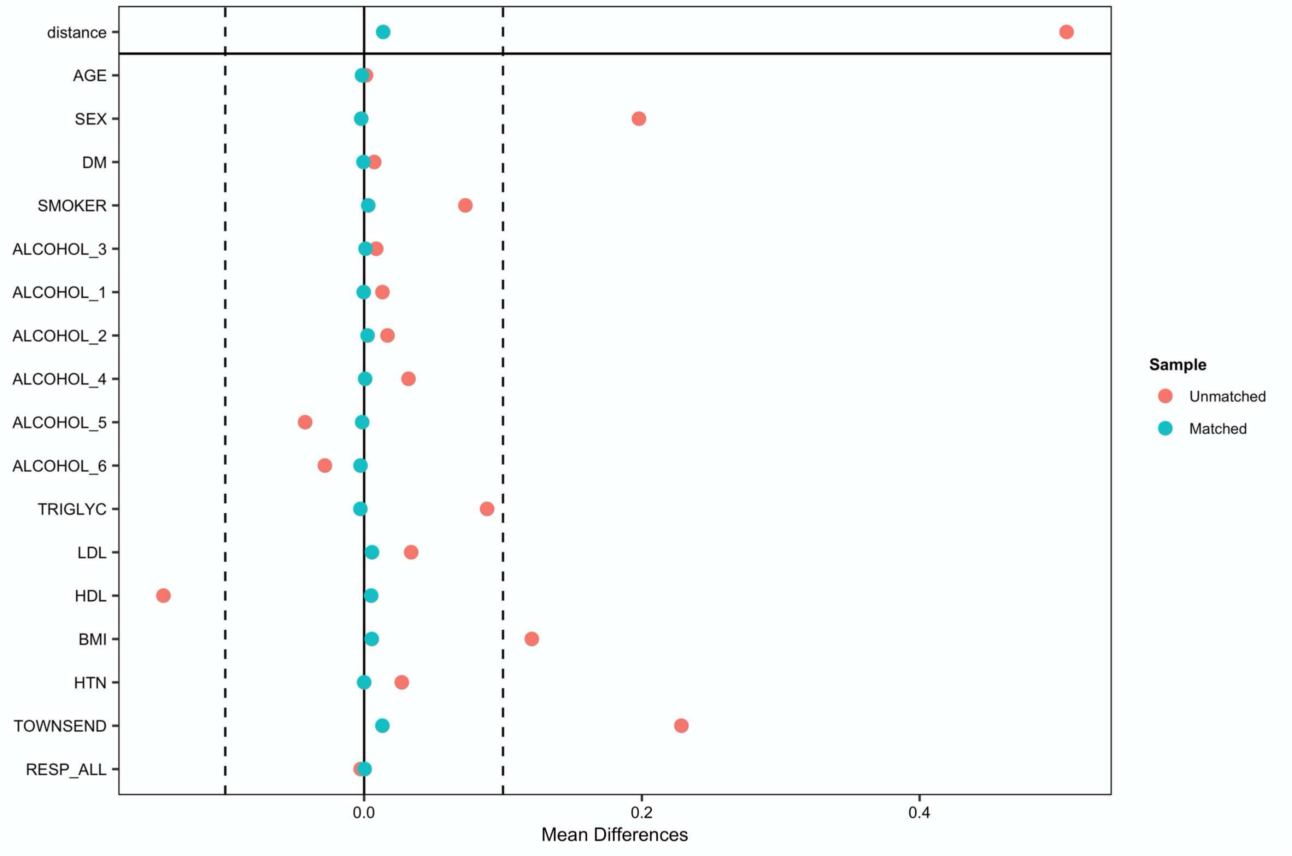

Supplement: Supplementary data [file oemed-2022-108670supp002.pdf]
